# Fabrication, Tensile Properties, and Photodecomposition of Basalt Fiber-Reinforced Cellulose Acetate Matrix Composites

**DOI:** 10.3390/polym13223944

**Published:** 2021-11-15

**Authors:** Yuxi Shen, Alia Gallet-Pandellé, Hiroki Kurita, Fumio Narita

**Affiliations:** 1 Department of Materials Processing, Graduate School of Engineering, Tohoku University, Sendai 980-8579, Japan; shen.yuxi.t1@dc.tohoku.ac.jp; 2 Department of Frontier Sciences for Advanced Environment, Graduate School of Environmental Studies, Tohoku University, Sendai 980-8579, Japan; alia.gallet.pandelle.q7@dc.tohoku.ac.jp (A.G.-P.); kurita@material.tohoku.ac.jp (H.K.); 3 Department of Materials Science and Engineering, MATEIS, CNRS UMR5510, INSA-Lyon, Université de Lyon, CEDEX, 69621 Villeurbanne, France

**Keywords:** mechanical testing, natural fibers, polymer matrix composites (PMCs), strength, environmental degradation

## Abstract

Cellulose acetate (CA) is widely used as an alternative to conventional plastics because of the minor environmental impact of its decomposition cycle. This study synthesized five-layer environmentally friendly composites from CA bioplastic and basalt fibers (BFs) to produce a high-strength marine-biodegradable polymer. Maleic anhydride-grafted polypropylene (PP-g-MAH) was mixed with CA as a surface-active agent (SAA) to understand the effect of surface treatment on the mechanical properties of the composite. Tensile tests and scanning electron microscopy were conducted to observe the fracture surfaces. The ultimate tensile strength (UTS) of the BF/CA composite increased by approximately a factor of 4 after adding 11 vol.% unidirectional BF. When the SAA was added, the UTS of the composite with 11 vol.% BF was multiplied by a factor of about 7, which indicates that the surface treatment has a significant positive effect on the mechanical properties. However, the improvement is not apparent when the added BFs are in a plain weave with a vertical orientation. A photodecomposition experiment was then conducted by adding TiO_2_. Observing the UTS changes of the CA and BF/CA composites, the effect of the photocatalyst on the decomposition of the materials was explored.

## 1. Introduction

In a context of diminishing resources and climate change, the need to shift to a more sustainable society implies changing the way materials are designed, used, and discarded. For example, fiber-reinforced polymers (FRPs), combining lightness with high strength and stiffness, have been used in various fields, including the aerospace, automotive, civil engineering, construction, and the medical equipment industries [1,2,3,4,5,6,7]. However, traditional FRPs, reinforced with glass or carbon fibers, use petroleum-based resins and raise end-of-life issues. Therefore, the demand for research and development into alternative composite materials with a minor environmental impact has been growing [8]. Green composites, i.e., bio-based resins reinforced with natural fibers, are made from renewable resources and may be biodegradable, reducing the carbon emission inherent to burning plastics [9,10].

Polylactic acid can be mass-produced in large commercial plants from starch, has a low market price, and has high strength and rigidity compared to other resins. Therefore, it is attracting considerable attention as a bio-based resin with applications as the matrix phase in green composites [11,12,13]. Additionally, polybutylene succinate is a new bio-based and biodegradable resin [14,15]. The development of a method to produce raw material succinic acid at a low cost using raw plant materials, such as sugarcane and corn, may explain this increase in interest [16]. Cellulose acetate (CA) is a modified natural polymer with many properties, such as high yield strength, biocompatibility, moderated hydrophilicity, excellent workability, and low price, making it one of the most essential organic esters derived from cellulose [17,18]. CA can be obtained via an esterification reaction using cellulose as the raw material, acetic anhydride as an acylating agent, and acetic acid as a solvent. According to the degree of substitution, the product of this reaction is divided into CA, cellulose diacetate, and cellulose triacetate. It is well known that the degree of acetylation affects the physical properties of CA. Highly acetylated CA has excellent mechanical properties and high flexibility. CA has often been reinforced using fibers to enhance its mechanical properties [19].

Although there are many varieties of natural fiber, they can be broadly classified into plant, animal, and mineral fibers. Recent works focus mostly on relatively high-strength plant fibers, such as jute [20,21], flax [22], sisal [23,24], and nanocellulose [25,26,27,28]. Conversely, there are relatively few examples of works investigating animal fibers. However, silk fiber, a typical animal fiber, is highly ductile (unlike plant fibers) and is expected to be a reinforcing fiber that contributes to increasing the impact resistance of composite materials [29]. Basalt fiber (BF) is formed from basalt rocks through melting and drawing processes [30]. Basalt being produced via the eruption of magma is resistant to high temperatures and exhibits strong chemical stability [31]. BF is expected to reinforce new environmentally friendly fiber materials because of its remarkable tensile properties. For example, the Young’s modulus of BF (90 GPa) is higher than that of glass fiber (76 GPa) [32].

Contrary to prior incorrect conclusions, the biodegradability of CA is recognized today [33]. In 1972, Potts et al. [34] investigated polymer degradation by exposure to fungi and concluded that CA is not biodegradable under that condition. Northrop and Rowe [35] studied the decomposition of CA fibers in moist soil in 1987. The degradation was already visible after 2 months, and the fibers were destroyed after 4 to 9 months. Other studies have confirmed the biodegradability of CA. Buchanan et al. [36] assessed the CA biodegradation in two aerobic conditions.

On the one hand, CA fibers placed in an in vitro enrichment culture were degraded within 2 to 3 weeks. On the other hand, the degradation of CA films suspended in an activated sludge waste treatment system was slower, with significant deterioration of the samples visible only after 10 weeks. These results show that CA is potentially biodegradable and that the speed of degradation depends on the environment.

Photodegradation, along with biological degradation, is an essential pathway for the degradation of CA materials in the environment. These materials exhibit radical formation initiated by the absorption of light. Free radical generation and CA photodegradation by ultraviolet (UV) irradiation have been reported in previous studies, despite CA’s high photostability. Although CA is made from raw materials rich in α-cellulose, these materials contain impurities that may be responsible for light absorption in the far-ultraviolet region (wavelengths shorter than 280 nm) [37,38,39]. CA absorbs light at about 260 nm due to ketonic carbonyl groups [33]. Therefore, the photodegradation of CA in the ambient environment seems to be very slow. One effective way to accelerate the degradation of polymeric materials in the natural environment is to add photosensitizers to the polymers.

The most common photocatalyst is titanium dioxide (TiO_2_). The photocatalyst TiO_2_, with a particle size of 0.2–0.3 μm, is often used as a deodorizer and whitening agent for textiles and polymer materials. TiO_2_’s photoactivity has been widely studied [40,41,42,43]. When anatase crystals are irradiated with near-ultraviolet light (<390 nm), electrons are excited from the valence band of the conducting band, and oxygen can be reduced by electron transfer. In addition, holes remaining in the valence band may oxidize water adsorbed on the surface, producing various radicals. These radicals can initiate any degradation of the surrounding polymer by hydrogen abstraction with subsequent radical chain reactions of the polymer, especially at high temperatures when the activation energy for peroxide cleavage is achieved.

In this study, unidirectional and two-dimensional BF fabric-reinforced CA matrix (BF/CA) composites, both with and without a surface acting agent, were fabricated to improve the mechanical properties of CA via the use of BF fabrics. The tensile properties of the BF/CA composite were evaluated and discussed. The fracture surface of the BF/CA composite was observed using a scanning electron microscope (SEM). Biodegradable CA composites with added TiO_2_ were then manufactured, and their ultimate tensile strength (UTS) was evaluated. The effect of three variables on the synergistic strengthening was considered: the amount of TiO_2_ in the composite, the decomposition time, and the different decomposition environments. Finally, the composites were subjected to mechanical deformation to investigate the change in their tensile properties.

## 2. Experimental Procedure

### 2.1. Materials

CA pellets (EC210, Daicel Corporation, Osaka, Japan) and BF fabrics (BWUD-200 and BWP-108, Zhejiang GBF Basalt Fiber Co., Ltd., Dongyang, China) were prepared as starting materials. The CA density and melt flow rate are 1.28 g/cm^3^ and 14 g/10 min, respectively. The BF diameter is 9 μm, and 500 fibers are bundled as 1 yarn. The thicknesses of BWUD-200 and BWP-108 are 0.28 mm and 0.15 mm, respectively. BWP-108 is a plain fabric. In addition, acetone (Fujifilm Wako Pure Chemical Corporation, Osaka, Japan), maleic anhydride graft polypropylene resin (PP-g-MAH; MG400-EM, Riken Vitamin Co., Ltd., Tokyo, Japan), and TiO_2_ powder (ST-01, Ishihara Sangyo Kaisha, Ltd., Osaka, Japan) were also prepared. The diameter of TiO_2_ powder is 7 nm.

### 2.2. Experimental Design

The effect of BF orientation and volume fraction on the mechanical properties of BF/CA composites was considered by testing samples reinforced with unidirectional BF and vertical plain-weave BF for various fibers/matrix ratios. Additionally, because there are fewer surface-active groups on the BF surface, an active agent with more –OH groups was added as a surface treatment to understand the effects of surfactants on the properties investigated. The following experiment was designed to investigate the effect of three variables: the amount of TiO_2_ photocatalyst, the decomposition time, and the decomposition environment. By testing the CA and BF/CA composites both with and without the addition of TiO_2_, the effect of TiO_2_ on the strength of the composites was observed. The effects of UV light and water were both evaluated with and without the addition of TiO_2_.

### 2.3. Fabrication of the CA Layer

**Figure 1:**
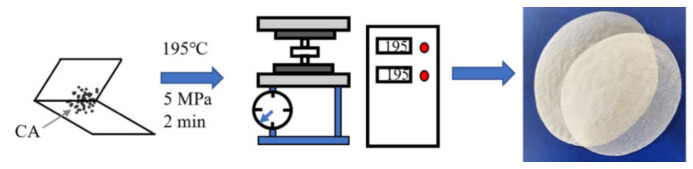
Fabrication process of the CA layer.

The CA layer was prepared first. Particulate CA was placed on kitchen baking paper and hot-pressed at 195 °C and 5 MPa for 2 min. After cooling, a semitransparent and smooth CA layer was obtained (Figure 1).

### 2.4. Fabrication of the CA-SAA

**Figure 2:**
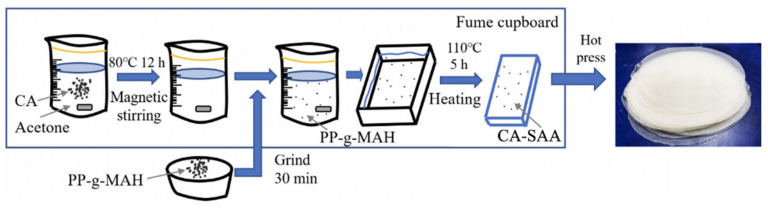
Mixing process of CA and SAA.

PP-g-MAH is fabricated by grafting maleic anhydride onto polypropylene. It is often used as a surface-active agent (SAA) to improve the adhesion and compatibility of several materials with different polarities since PP-g-MAH has maleic anhydride as a polar group on a non-polar polypropylene backbone. Therefore, PP-g-MAH was added into CA to improve the interfacial strength between CA and BF, as shown in Figure 2. CA (20 wt.%) was first dissolved in acetone at 80 °C and stirred mechanically for 12 h. After the CA is completely dissolved, forming a viscous gel, SAA (2 wt.%), which had been ground for 30 min, was added. The viscosity of the CA allowed the SAA to be dispersed well. The mixture was then placed on a heating plate at 110 °C for 5 h to remove the acetone and obtain the final CA-SAA product.

### 2.5. Fabrication of the CA–SAA–TiO_2_ Layer

To prepare the CA–TiO_2_ mixture, TiO_2_ (1 wt.%) was added to CA and stirred for 10 min until it became a milky white suspension. CA (20 wt.%) was dissolved in acetone at 80 °C and stirred mechanically for 12 h. When the CA was completely dissolved, forming a viscous gel, the TiO_2_ was added. The viscosity of the CA allowed for the TiO_2_ to be dispersed well. The mixture was then placed on a heating plate at 110 °C for 5 h to remove the acetone and obtain the CA–TiO_2_ product.

**Figure 3:**
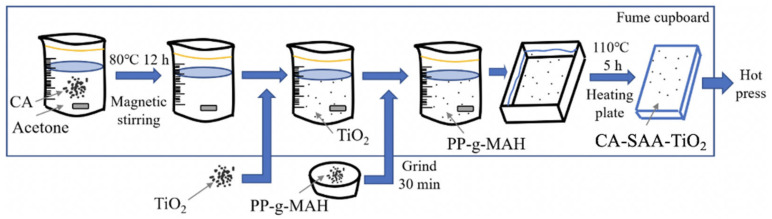
Mixing process of CA, SAA, and TiO_2_.

In some cases, SAA (2 wt.%) that had been ground for 30 min was also added to the CA–TiO_2_ product. The mixture was then placed on a heating plate to obtain the final CA–SAA–TiO_2_ product (Figure 3).

### 2.6. Fabrication of BF/CA Composites

**Figure 4:**
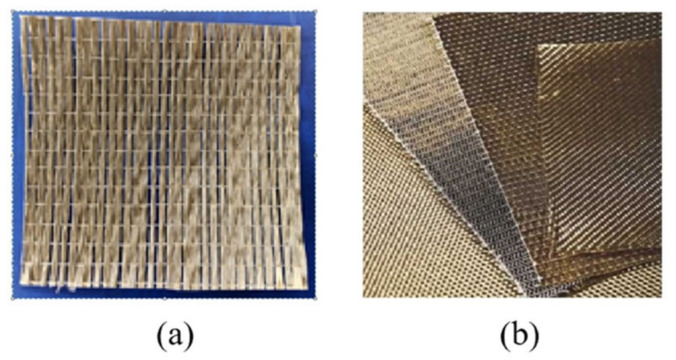
Two BF types: (**a**) unidirectional BF and (**b**) vertical plain-weave BF. White fiber in (**a**) is a thin polymer thread to maintain the BF sheet shape.

**Figure 5:**
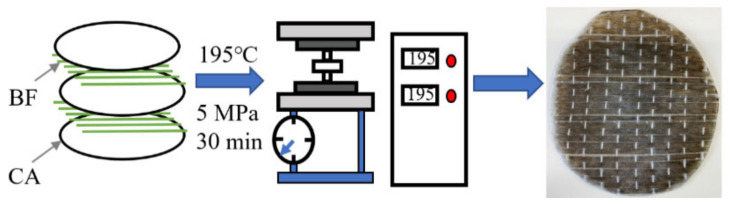
Fabrication process of BF/CA composites: White fiber is a thin polymer thread to maintain the BF sheet shape.

Two types of BF were used in this work (Figure 4). The BF and CA layers were then alternately stacked and subsequently heated and pressed at 195 °C and 5 MPa for 3 min (Figure 5). Different BF-enhanced CA composites were manufactured by adding 0, 4, 7, 9, and 11 vol.% unidirectional BF. The vertical plain-weave BFs were difficult to disperse in solvent, unlike the unidirectional BF. Hence, different numbers of layers were used to alter the volume fraction. Layers 1, 2, or 3 of vertical plain-weave BFs were added to the CA or CA–SAA layers to form 4, 7, or 11 vol.% reinforced composites, respectively.

### 2.7. UV Decomposition Test

Eight groups of samples were characterized: (1) CA, (2) CA–TiO_2_, (3) CA–SAA, (4) CA–SAA–TiO_2_, (5) CA with 11 vol.% unidirectional BF, (6) CA–TiO_2_ with 11 vol.% unidirectional BF, (7) CA–SAA with 11 vol.% unidirectional BF, and (8) CA–SAA–TiO_2_ with 11 vol.% unidirectional BF. Each of the eight sample groups was subjected to three different environments. Environment (a) is a setting with no UV light exposure and no water; therefore, this group represents a scientific control. Environment (b) is an environment with UV light exposure but no water to observe the effect of UV light on the decomposition process and tensile properties of the CA and BF/CA composites. Environment (c) is an environment where the samples are exposed to both UV light and water to observe the effect of water on material degradation. The wavelength of the UV light used was 154 nm. The 8 sample groups were taken out after 5, 10, and 15 days and tested to observe the effect of the different surroundings on the strength as a function of time. Five specimens were prepared for each test condition.

### 2.8. Tensile Testing

Sixteen groups of samples were characterized: (1–5) CA with 0, 4, 7, 9, and 11 vol.% unidirectional BF; (6–10) CA–SAA with 0, 4, 7, 9, and 11 vol.% unidirectional BF; (11–13) CA with 4, 7, and 10 vol.% vertical plain-weave BF; and (14–16) CA–SAA with 4, 7, and 10 vol.% vertical plain-weave BF. The tensile properties of UV decomposition test specimens were also evaluated. Tensile tests were conducted using a Universal Testing Machine (AGS-X 10KN). Specimens with a length of 82.3 mm and a width of 8.2 mm were cut. For each sample type, a set of five specimens were tested until failure at a rate of 2 mm/min according to the ASTM D 3039 standard [44]. The tensile properties were evaluated from the tests, in which the specimens failed between the gage marks. After tensile failure, small samples containing the fracture surface were cut from the tensile specimens. These samples were sputter-coated with gold and observed via SEM under a high vacuum using an acceleration voltage of 1 kV.

## 3. Results and Discussion

### 3.1. Tensile Behavior of Unidirectional BF-Reinforced Composites

**Figure 6:**
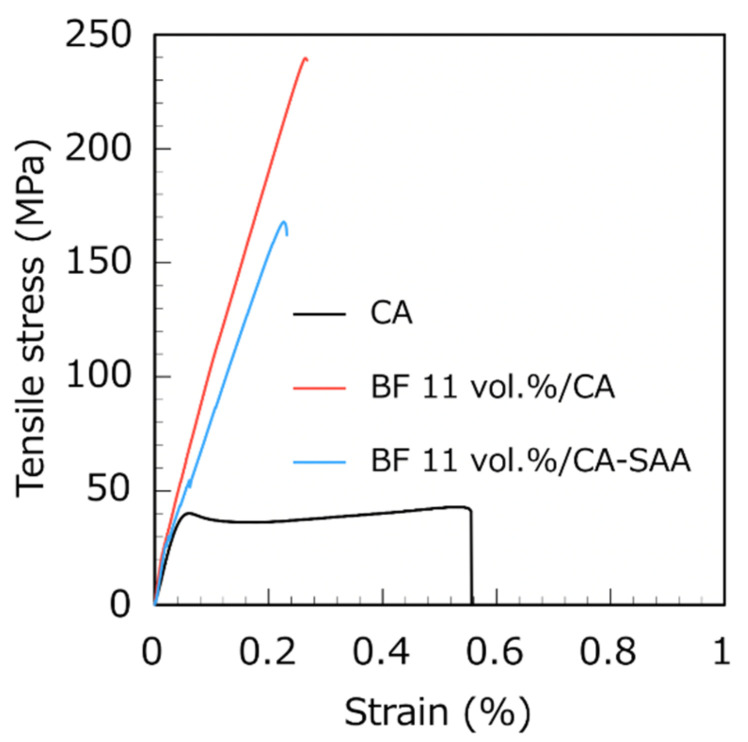
Stress–strain curves of pure CA and unidirectional BF-reinforced composites with 11 vol.% BFs, with and without the addition of SAA.

Figure 6 shows the typical stress–strain curves for the pure CA and the unidirectional BF-reinforced composites with 11 vol.% BFs, with and without the addition of the SAA. Pure CA is used as the control group and exhibits the intrinsic mechanical characteristics of the polymer. The middle of the pure CA samples became thinner with increasing force; the samples exhibited yielding behavior for a long time before it finally broke at the thinnest point of the samples. The samples showed brittle fracture under stress after adding unidirectional BFs or after the surface treatment with SAA.

**Figure 7:**
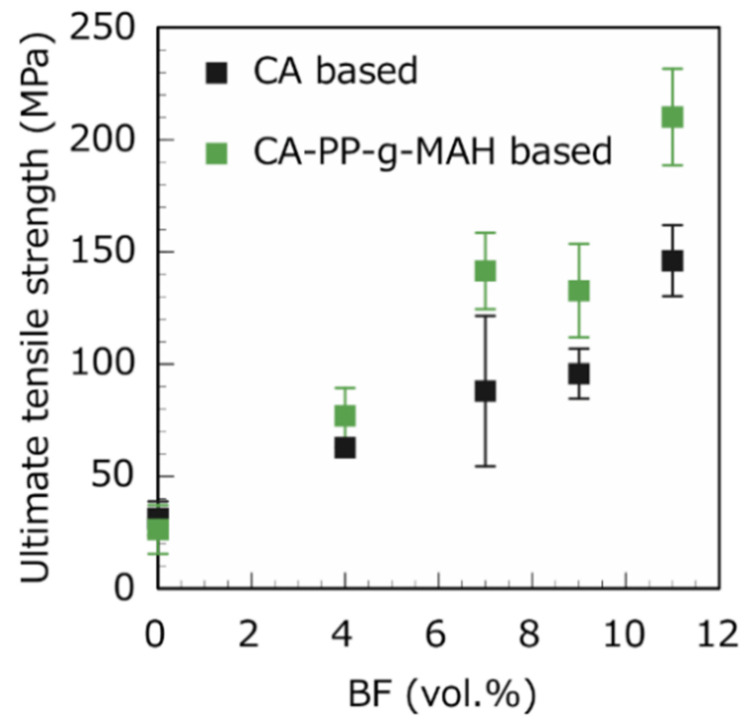
Different BF volume fractions of unidirectional BFs affect the UTS of BF/CA composites and BF/CA–SAA composites.

**Figure 8:**
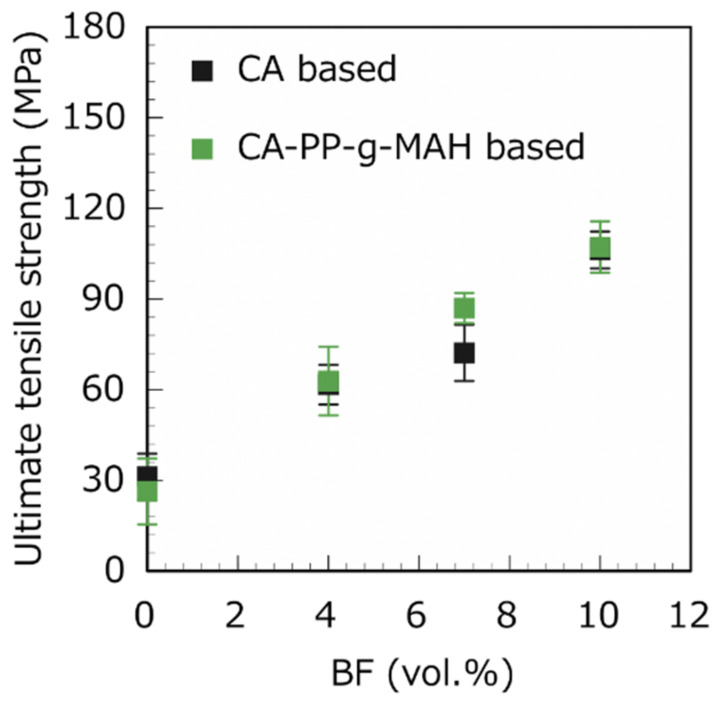
Different volume fractions of plain-weave BFs affect the UTS of BF/CA composites and BF/CA-SAA composites.

Figure 7 shows the dependence of the UTS on the BF volume fraction for the CA and CA–SAA unidirectional BF-reinforced composites. Increasing the unidirectional fiber content increases the UTS of the composites. The UTS of the BF/CA composite increased by approximately a factor of 4 after adding 11 vol.% unidirectional BFs, whereas that of the BF/CA–SAA composite increased by a factor of approximately 7 after adding 11 vol.% unidirectional BF. Increasing the vertical plain-weave BFs in the composites also improved the UTS (Figure 8). However, the effect of surfactants was minor for plain-weave fibers compared to unidirectional BFs. One should also notice that, for a given fiber content, the UTS is higher when using unidirectional fibers. For instance, the BF/CA–SAA composites with 11 vol.% of fibers showed a strength of about 210 MPa for unidirectional fibers, compared to around 110 MPa when using plain-weave fibers, i.e., the strength was almost doubled. This result indicated a relatively poor adhesion between the plain-weave BFs and the CA matrix.

Table 1 shows a comparison of the UTS and modulus of the 11 vol.% unidirectional and plain-weave BF/CA–SAA composites with other green composites taken from the literature [45,46,47]. The data regarding the CA layer are also presented. On the one hand, one can compare the tensile properties of the BF/CA–SAA samples of the present work with other cellulose acetate bio-composites but reinforced with different types of natural fibers. Two studies using 28 vol.% woven jute fibers and 40% hemp fibers, respectively, reported a tensile strength around 58 MPa and a Young’s modulus of 5.3 and 6.0 GPa, respectively. The modulus values are comparable to the one of plain-weave BF/CA-SAA with 11 vol.% fibers but the strength is halved. This difference might be explained by the higher tensile properties of basalt fibers, which are mineral natural fibers, compared to plant fibers. On the other hand, Samper et al. [47] investigated another combination of environmentally friendly composite materials using mineral fibers, that is, an epoxidized linseed oil matrix reinforced with 46 wt.% slate fiber fabrics. Compared to the 11 vol.% unidirectional BF/CA–SAA sample, which is the most performant sample of this study, the strength is multiplied by a factor of 1.5 and the elastic modulus is doubled.

### 3.2. Effect of Surface Treatment on the Tensile Properties

For a given BF volume fraction, except for the samples with 0 vol.% BF, the UTS of the CA–SAA composites was higher than that of the CA composites. A possible explanation is that the many active –OH groups of PP-g-MAH improved the bonding at the interface between the CA matrix and BFs. On the other hand, in the cellulose acetate samples without fibers, the SAA might have broken the bonding between the CA monomers during the preparation of the CA, resulting in the lower UTS observed.

### 3.3. SEM Observation of the Fracture Surfaces

**Figure 9:**
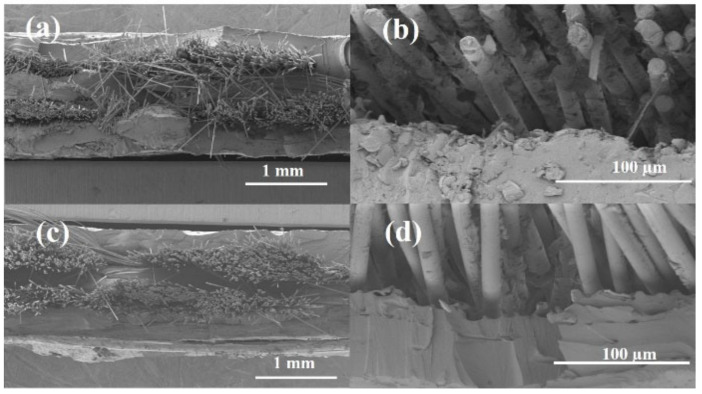
SEM images of the fracture surfaces of unidirectional BF (11 vol.%) reinforced composites: (**a**) BF/CA at low magnification; (**b**) the interface between the BFs and the CA matrix at high magnification; (**c**) BF/CA–SAA at low magnification; (**d**) the interface between the BFs and the CA–SAA matrix at high magnification.

Observation of the fracture surface showed that the main material failure route was fiber fracture. Figure 9a,b shows the SEM images of the fracture surfaces of the BF/CA composites at low and high magnification, respectively. Figure 9c,d shows the SEM images of the BF/CA–SAA composites at low and high magnification, respectively. As shown in Figure 9b or Figure 9d, the fibers were pulled out from the edge of the composites. The smooth surface of the BF limits the interface cohesion between CA or CA–SAA and BF.

### 3.4. Tensile Strength of CA Composites after UV Decomposition

**Figure 10:**
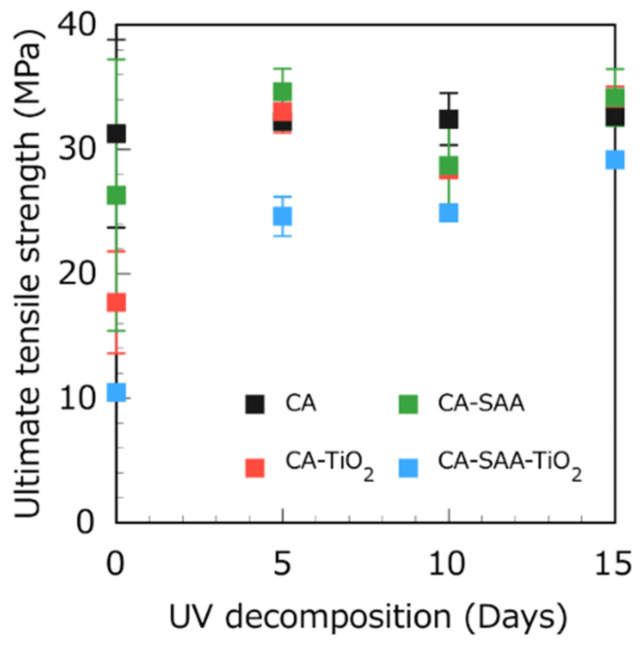
UTS of CA layers with different additives after UV decomposition during 0, 5, 10, and 15 days.

The UTS of the composites was measured after 0, 5, 10, and 15 days of exposure to UV light. Figure 10 shows the UTS of the CA layer with different treatments. First, in the samples without UV exposure (0 days), adding TiO_2_ and SAA reduced the UTS (in comparison with pure CA) because the particle radius of TiO_2_ is on the nanoscale (7 nm), which is much smaller than the generally used 200 to 300 nm. Therefore, it is hypothesized that the surface energy of the particles is too large, and they thus agglomerate too easily. After 5 days, the UTS of each CA sample was improved. The cause of this increase has not yet been established. 

**Figure 11:**
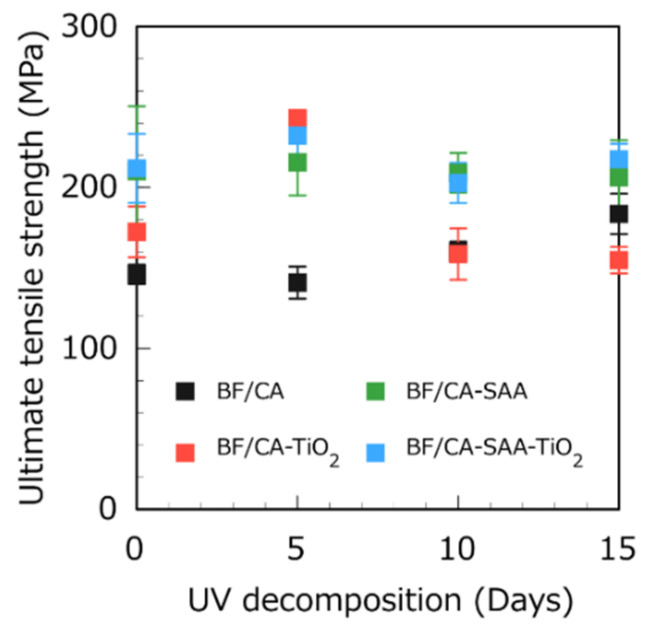
UTS of 11 vol.% BF/CA composites with different additives after UV decomposition during 0, 5, 10, and 15 days.

Figure 11 shows the UTS evolution of different BF/CA composites with 11 vol.% of fibers regarding the UV exposure time. Overall, the change in the UTS of the BF/CA samples is relatively small. The UTS of the BF/CA-TiO_2_ composite was larger on day 5 than on day 0 but decreased significantly between day 5 and day 10. Therefore, a hypothesis can be made that adding TiO_2_ promotes the photodecomposition of CA and that the decomposition begins after 5 days of UV irradiation. The UTS remains stable during the whole photodecomposition experiment when SAA is added to the composites. Before UV exposure, on day 0, there was little difference in UTS between the four groups of BF/CA composites. With increasing UV irradiation time, the difference between the samples with and without SAA became apparent. For the two groups with SAA, the UTS did not change significantly between day 5 and day 15. Further experiments are required to understand these UTS variations.

## 4. Conclusions

This study manufactured biodegradable CA layers and CA composites with added BFs, and their tensile properties were evaluated. The effects of the BF volume fraction, BF orientation, and surfactant were investigated. Adding BFs in the polymer matrix increased the UTS of the composites. The UTS of the BF/CA composite increased approximately by a factor of 4 after adding 11 vol.% unidirectional BFs, whereas that of the BF/CA–SAA composite increased by a factor of approximately 7. Adding surfactants also increased the UTS of the samples by improving the fiber/matrix adhesion. The influence of different components on the photodecomposition of the BF/CA composites was measured via tensile tests after UV irradiation of varying durations. TiO_2_ addition affected the UTS of BF/CA composites. However, further experiments are required to understand these UTS variations. Nevertheless, the BF/CA composite is likely a promising structural material in the fishery.

## Figures and Tables

**Table 1:** Comparison of the UTS and modulus of green composites.

Sample	UTS (MPa)	Young’s Modulus (GPa)	
CA	35	1.9	This study
Unidirectional BF/CA–SAA (11 vol.%)	220	11	This study
Plain-weave BF/CA–SAA (11 vol.%)	110	7	This study
Jute fibers/CA	58	5.3	[45]
Hemp fibers/CA	58	6.0	[46]
Slate fibers/Epoxidized linseed oil (ELO) matrix	330	22	[47]

## Data Availability

The data presented in this study are available on request from the corresponding author.
